# Medico-Historical Account of the Western Coast of Africa

**Published:** 1832-04-01

**Authors:** 


**A PRACTICAL MEDICO-HISTORICAL ACCOUNT OF THE WESTERN COAST OF AFRICA; EMBRACING A TOPOGRAPHICAL DESCRIPTION OF THE SHORES, RIVERS, AND SETTLEMENTS, WITH THE CAUSES, SYMPTOMS, AND TREATMENT OF THE FEVERS AND OTHER DISEASES OF WESTERN AFRICA.** By *James Boyle*, M.R.C.S. Colonial Surgeon to Sierra Leone, Surgeon R.N. &c. Octavo, pp. 424. Highley, Dec. 1831.

It is not a little extraordinary, as Mr. Boyle remarks, that no work on the diseases of Africa should yet have emanated from the medical press of this country, so fertile in all kinds of publications on very inferior subjects. Yet Africa is the grave of Europeans, and especially of the English, beyond that of any other colony, in proportion to extent or population. Our travellers, medical and non-professional, have penetrated into the wilds of that barbarous and deadly quarter of the world—while our soldiers, sailors, and merchants have been swept from its pestilential shores by sickness and death; yet no medical work has been published on the nature of the diseases, or the means of guarding against them. But however destructive may be the climate of Africa, there is reason to believe that its terrors have been sometimes exaggerated, and that men of talents and acquirements, in whatever calling, have generally been intimidated from serving in that colony. Even those who have struggled strenuously to brave the dangers of the climate, have too often been hastened to the grave by the foreboding and anticipations of sickness or death.

It is hardly possible to give up intercourse with the African Coast, so extensive is our trade in gold dust, ivory, wax, hides, &c. besides timber of a valuable kind. It behoves us, therefore, to make the best of it, by lessening the mortality if possible.

Mr. Boyle had fair opportunities for observing the diseases of Western Africa, both at sea, and as colonial surgeon during five or six years there. The records, also, of our naval and military medical practitioners have been liberally laid open to him, by Sir James Macgrigor and Sir William Burnett, from which documents the author has gleaned important information.

The work is divided into eight chapters, each embracing a distinct subject. The first chapter, occupying 70 pages of letter-press, is on the important subject of medical topography, and takes in a wide range, including the river Gambia, Sierra Leone, Bathurst Settlement, Rio Grande, &c. We shall only be able to give a few extracts from this highly-interesting portion of the work. The first is from the medical topography of Bathurst Settlement, the most northern of the British establishments, and the chief on the river Gambia. It is situated on the small island of St. Mary, commanding the navigation of the river, but badly adapted for the preservation of health. It consists of barren sand and tenacious mud, overgrown with jungle.

"The wet season is immediately preceded by tornadoes or thunder-storms, which, together with the harmatan, another wind peculiar to this coast, will be described under the head of Sierra Leone, as they are the most strongly felt at that settlement. The rains are tremendous, and swell the river so considerably, that, during their fall, almost the whole of the island is inundated. The part upon which the town is built escapes not, and but for the precaution taken in building the houses, many would be uninhabitable during this period. When the rains subside, and the river returns to its level, an immense deposit of vegeto-animal matter is left behind, the whole of which undergoes the process of decomposition, and, from being acted upon whilst in that state by the rays of an almost vertical sun, fills the air with noxious effluvia. This effluvia, combined with the remaining moisture and the high range of temperature, form the great cause of fever and of ague, which prevail during its existence to so lamentable an extent as to make this settlement appear a focus for those diseases and the grave of Europeans." 6.

### Sierra Leone.

This settlement, the principal one on the Western Coast of Africa, and about which we all have heard so much, is beautiful, or even romantic, to the eye, when first approached on a fine day; but deadly to the health when taken up as a residence.

"There are very few parts in the tropical world, which, at first sight, hold out more allurements, even to the experienced traveller, than Sierra Leone. Its splendid scenery, and its beautiful river, together with its extensive, commodious, and generally secure harbour, and pleasant-looking town and villages, are calculated to excite the most flattering hopes in respect of health and enjoyment, notwithstanding strong previous impressions to the contrary. On making Sierra Leone from the north, the mountains from which the peninsula was named, first excite attention. They are lofty, perpetually clothed, from their summits to their bases, in all the fertile gaiety of nature's verdant and richest scenery; and there is a pleasing and endless variety in the outline of their countless peaks and declivities. As the ship draws in with the shore signs of civilization appear and increase with rapidity, both in number and attractiveness. Freetown and the lately-formed villages in its neighbourhood at first shew like anomalous patches in the view, but, on a nearer approach, they add greatly to its beauty and its interest. When the ship has arrived just at that point of distance from which a person may see all the broad outlines and apparent characteristics of an extensive scene, without being able to discern the minute details, the effect is magnificent. On the left hand is the Bullom shore, low but covered with luxuriant and richly-coloured bush, an occasional palm and pullom tree, rising in graceful form above the neighbouring mangroves: in appearance it seems to embody the notions formed of fairy land, but its realities most sadly illustrate the folly of such dreams. The middle ground also occurs on the left hand, and it gives a variety to the view. In front are the spacious river, (extending farther than the eye can reach) and the north side of the peninsula, with its lofty mountains and Freetown, running to the water's edge, and surmounted by barracks, and protected by a handsome fort, and a coast forming numerous small and convenient bays from the town to its termination at the cape, which runs boldly into the sea. On the right is the Atlantic. That a scene, composed of such ostensible material features, is grand and imposing, may readily be supposed; but those who are ignorant of the peculiarities of a tropical clime, and its seductive influences on a stranger, can form no adequate notion of the character and extent of its actual power. For the moment home is forgotten, or if remembered, the remembrance is accompanied with a desire it should be situated in such a seeming paradise. In thus speaking of the view on arriving at Sierra Leone, we are supposing that settlement to be made on a fine clear day, when the atmosphere is bright and comparatively devoid of malaria, and the river runs its natural course, unswollen and free from discolouration. Should the arrival, however, happen at a different period, when the atmosphere is dense, oppressive, and fraught with deleterious exhalations, and the rains are deluging the face of the country, and at once augmenting the river and destroying its beauty, then Sierra Leone presents a very different appearance; there is little or nothing to excite a pleasing anticipation, but there are a world of causes for apprehension and for dread. The realities of the scene are of course unaltered, for the two periods are the property of the climate, and must be alike endured by the colonist, but the appearances present a melancholy and a fearful contrast." 25.

This settlement contains about 26,000 inhabitants, one-third of whom inhabit Freetown, the rest being distributed in the surrounding villages.

"The inhabitants of Freetown consist of Europeans, Maroons, Settlers or Nova Scotians, exiles from Barbadoes, discharged soldiers from the West India regiments, Mandingos, liberated Africans, and Kroo-men; while those of the villages consist almost exclusively of a very few Europeans, (as the managers and most of the church missionaries) discharged soldiers, and liberated Africans." 30.

The Europeans are about 120 in number, and, with few exceptions, adult males; women and children are rarely met with, as they are unable to resist the deleterious influence of the climate—at least in Freetown; for in the villages, which are more healthy, they stand a better chance. It appears from Mr. Boyle, that the influence of climate is augmented by the imprudent habits of the colonists. They are too frequently loose, careless, and dissipated.

"It would not be difficult to assign many other reasons for much of the suffering notoriously endured by Europeans in this settlement; but, after all, the great leading cause would be found to be the intrinsic quality of the climate of that region. Wholly and successfully to oppose that cause human ingenuity can hope to devise no adequate plan, but many suggestions might be offered calculated to meliorate its influence. It may be sufficient to remark in this place, that a moral life, freedom from fear, cheerfulness, moderate exercise, and particular care in avoiding the extremes of the atmosphere, are absolutely necessary to allow the English constitution in this climate a fair chance of sustaining life. To the practice of these auxiliaries to health, Sierra Leone offers no preventives; but, on the contrary, many facilities, which are to be found in the construction of Freetown and in the mountains." 31.

Mr. Boyle gives an excellent description of the seasons of Sierra Leone, dividing them for convenience into the dry and the wet seasons. The dry commences about the end of September, its approach being usually announced by two or three tornadoes, which, though portentous in appearance, are hailed with joy by the inhabitants, as harbingers of health. We shall indulge our readers with a description of one of these elementary conflicts.

"A violent tornado appears to strangers a most appalling visitation, and produces an extraordinary effect upon their feelings. It consists of successive flashes of most vivid lightning, tremendous shocks of thunder rapidly and alarmingly reiterated, impetuous gusts of wind, and deluging rain. This terrific combination of the elements sweeps along the whole of the coast under consideration; but it occurs with peculiar violence, on what is called the windward coast, especially at Sierra Leone. Its denomination is derived from the Portuguese, it being a corruption of the word *Trovado*, which means thunder-storm. Its approach is first discernible by the appearance of a small, clear, silvery speck at a high altitude in the heavenly expanse, which increases and descends towards the horizon with a gradual and slow, but visible motion. In its descent it becomes circumscribed by a dark ring, which extends itself on every side, and as soon as the silvery cloud approaches the horizon, veils it in impenetrable gloom. At that moment the elements seem to have ceased their operations, and the very functions of nature to be paralyzed; the atmosphere appears to be deprived of the spirit of vitality, and a sensation of approaching suffocation pervades and oppresses the physical system. The mind is wrapt in awe and suspense, but the latter is speedily relieved by the dark horizon being suddenly illumined by one broad blaze of electric fluid; peals of distant thunder then break upon the ear and rapidly approach and increase in frequency and violence till the shocks become appalling; when the thunder is at its loudest a tremendous gust of wind rushes with incredible, and often irresistible vehemence from the darkened part of the horizon, not rarely in its course carrying away roofs of houses and chimney-tops, blowing down or up-rooting trees and laying the stiffest and largest ships on their beam-ends, or sinking them, whether under-weigh or at anchor; and to that succeeds a furious deluge of rain, which falls in one vast sheet rather than in drops, and concludes this terrible convulsion." 41.

By this atmospheric commotion noxious exhalations, before confined to the neighbourhood of their sources, are swept away—the feelings are lightened—the weather becomes hot and clear—the inhabitants healthy. About Christmas the harmattans occur, and last for some weeks. They blow from the eastward. They impregnate the air with an impalpably-fine sand, and one very annoying to the seasoned Europeans—more so than to those newly arrived. During these dry winds the furniture splits—fluids quickly evaporate. They induce coughs, colds, and a species of irregular intermittent fever among the old settlers. About the beginning of March a tornado usually ushers in the rainy season, which is at its acmé in July, and subsides in September.

Mr. Boyle winds up the medical topography of the African coast with a meteorological diary of every day in a whole year, by which it appears evident that the temperature of Sierra Leone and other parts is greater, upon the whole, than that of Madras or Calcutta, while the rains are more frequent, and the terrestrial circumstances not at all less unfavourable to health, than in either the Eastern or Western Hemisphere. The author concludes this portion of the work with some very judicious observations on hygiene, as applicable, in an especial manner, to Sierra Leone. It appears from Mr. Boyle, that the greatest source of illness at the abovementioned town is the Bullom swamp, situated in its vicinity. The author proposed some time ago, to Government, that a certain number of people, chiefly liberated African soldiers, should be located on that part of the swamp which is most contiguous to Freetown, in order to cultivate it, to plant it with trees, and thus to form a kind of defence against the malaria wafted by the winds to the capital of the colony. This corresponds with Italian hygiene, as may be seen in another part of our present number. We do not expect, however, that Government will attend much to matters of this sort under existing circumstances. It is too much occupied with matters of more vital importance. The colonists must look to themselves, as the colony of Great Britain itself was obliged to do, when the Roman Empire was threatened and environed with dangers at the centre!

### Chapter the Second.

In this chapter, Mr. Boyle takes up the subject of the climatorial, or

BILIOUS REMITTENT FEVER. The author considers it of great importance to discriminate between the climatorial fever and the "African local bilious remittent fever," which discrimination, unfortunately, cannot always be made by *symptoms* alone. Various other circumstances, of an etiological nature, must be taken into consideration.

"Sol-lunar influence is powerful in the production of fever on the western coast of Africa, and indeed in all parts between the tropics. Many instances have been known of men, whilst at work under the rays of the sun, dropping down, as if shot; and that, without any previous threatening symptom or habit of indiscretion; and also men, who, to avoid the closeness sometimes experienced in sleeping between decks, have slept on the upper deck, without the knowledge of the officer on watch, thus, exposing themselves to the apparently harmless beams of a brilliant moon, have often been known to be suddenly affected with fever. The rapidity of the latter attacks precludes the thought that they were attributable to damps or dews that might be falling in the night, and which, indeed, are also common causes of fever, but not so immediate in their consequences." 76.

Immoderate indulgence in ardent spirits is a predisposing cause of fever, and so is the opposite extreme—a sudden plan of abstinence through fear of the disease. Mr. B. avers that the absolute water-drinker is in as dangerous a predicament, when once seized with fever, as the absolute drunkard. We doubt this position. Where the fever occurs in situations abounding in filth, and badly ventilated, the disease, Mr. B. thinks, occasionally assumes a contagious character, especially in ships. The following is the symptomatology of this form of fever on the coast of Africa.

"This fever is *ordinarily* characterized by severe head-ache, pain at the pit of the stomach, retching or vomiting, with a costive state of the bowels: sometimes vomiting of green bile, great heat of skin, suffused eyes, and thirst; the tongue being generally more or less furred; usually, also, pains in all the joints, and tottering of the limbs. The pulse varies from 90 strokes in a minute to 120 or 140. On minute inquiry it will generally be found that the patient is suffering from severe pain in the back or loins; and if the face do not happen to be flushed at the time, it will have a livid hue, its features having a downcast appearance, and there being, in all probability, a dark areola around the eyes. Under these latter circumstances the pulse will be less developed; nor will the skin attain to so high a temperature. Most attacks will be preceded by a sense of chilliness, and attended with loss of appetite: and, in all, the greater part of the symptoms described will soon set in, and establish the true character of the disorder." 78.

Sixteen minutely-detailed cases, from his own and several other medical officer's journals are given, in order to delineate all the various features of the fever. These will be found to be valuable references for the surgeon on the coast of Africa, when surrounded by fever cases.

TREATMENT. From the cases alluded to it appears that, generally speaking, patients labouring under climatorial fever, on the coast of Africa, do not bear bleeding so well as in some other countries, and that the blood does exhibit the usual inflammatory phenomena. Venesection must, therefore, Mr. B. thinks, be practised with precaution, especially when the local endemic fever is mixed with the common climatorial disease.

"Still the result of considerable experience upon the coast, directly and indirectly, is, that blood-letting, with the above precautions, in such cases as appear to have a perfectly simple origin, that is, such as are altogether independent of local exposure, is, when the symptoms indicate increased arterial action, the first measure for adoption; the effect produced regulating the quantity to be taken away. Increased vascular action being reduced, the next object is to cause a slightly increased flow of saliva, by means of mercury. The ordinary, and, indeed, the better mode of administering this medicine, in the form of disease now under consideration, is to give large doses of calomel at the commencement, by which the bowels will be freely, but not very frequently moved; and by which, also, the system will be the more readily brought under the influence of that medicine. The bowels thus evacuated, two grains of calomel, with one-eighth of a grain of opium, may be given every two hours, until slight salivation and a remission of the febrile symptoms shall have taken place; when one grain of blue-pill, and two of quinine, in the form of pill, may be substituted, and repeated at the same intervals as that of the calomel and opium. After that, the blue-pill is to be gradually lessened, rather than suddenly withdrawn, as an abrupt cessation of salivation might be attended with fatal results.

A slight flow of saliva having once taken place, it should be kept up for a few days without cessation; and when a perfect remission of the febrile symptoms has been established, the blue-pill is to be altogether withdrawn, and equal quantities of rhubarb and quinine (say two grains of each) are to be given thrice daily until convalescence shall have fully taken place. The patient, during the period of convalescence, must be somewhat restricted in food and drink, and that rather in quantity than kind; for, strange as it may appear, it is a fact, that a moderate indulgence in the gratification of a desire, on recovering from this fever, is much less injurious than a total denial of the patient's wishes.

If the first abstraction of blood from the arm does not sufficiently subdue the febrile excitement, the local abstraction of blood, by means of cupping or leeches, from the seat of pain, may be adopted as the safest and most judicious practice; and if, after the use of that remedy, pain or mental aberration continue, or set in, the application of a blister to the neighbourhood of the affected part may, with propriety, be had recourse to. These measures are to be repeated according to the intensity of the disorder.

If there be great sickness of stomach, medicine by the mouth will have but little effect until leeching or blistering, or both, shall have been practised over the region of that organ; and, that effected, an occasional drachm dose of soda, dissolved in plain water, will, in all probability, have a powerful effect in restraining the gastric derangement. At such a time, mercurial frictions, instead of the administration of mercury by the mouth, will be proper; and small occasional doses of camphor mixture, with Tr. hyosciami, will contribute to quiet the stomach, to produce a diaphoresis, and to soothe the nervous system generally. If collapse take place, as occasionally happens in the advanced stages, ammonia should be added to the latter medicine; but, immediately re-action occurs from such combination, the ammonia is to be withdrawn, and quinine, in some form or other, to be administered. The camphor mixture, &c., may be continued, in order to keep up a certain action in the circulatory system. During the deranged state of the stomach alluded to, enemas will generally be found preferable to purgatives by the mouth; but when a purgative by the mouth is decided on, that should be calomel alone, followed by a magnesia draught. The quantity of calomel must, of course, depend on the object in view. If the intention be to purge and salivate, a large dose (a scruple, for instance), must be administered; but, if to purge with activity, without effecting salivation, be desired, then five or six grains, followed by a seidlitz powder, or two or three drachms of the phosphate of soda (tasteless salts, as they are commonly called,) will best answer the purpose.

As to bark, it is generally hurtful, if given in substance; indeed, it seems matter for surprise, how former writers and practitioners managed to get their patients to swallow some ounces of powdered bark in the course of the day. Modern stomachs would loathe or reject a few grains in such fever cases as have been under consideration.

The warm bath is an important remedy in the commencement of the fever; but, after that period, it is a remedy of questionable efficacy, owing to the debility attendant upon its repeated employment, and the danger of the patient's catching cold, when the mental powers are, in all probability, aberrated, and the physical faculties in a state of extreme exhaustion. After the first employment of the bath, and when the fever is at its height, and the patient has a hot, dry skin, the regular application of cold affusion of vinegar and water to the palms of the hands and soles of the feet, those parts being always the most affected with heat and tingling, will be found alike grateful and beneficial.

The use of antimonials of every description is to be deprecated, as they never fail to increase that irritability of stomach so commonly experienced, and so difficult of control, in this disorder.

When the mouth is decidedly under the influence of mercury, the febrile action positively subdued, and nothing but debility to contend with, wine, at first in small quantities, may, with propriety, be given. The period for this indulgence will generally shew itself on the seventh, eighth, or ninth day. This is to be looked upon as a general rule, rather than as one without an exception; for it will sometimes happen, that unexpected collapse, and sinking of the pulse, will take place, and call for the temporary use of wine. In such case, good old Madeira seems to answer the purpose best; but it is to be borne in mind that, when re-action takes place under the administration of wine, or any other powerful stimulant, the stimulant is to be withdrawn, unless the patient shall have progressed to the state alluded to above, when its use may be continued with propriety." 117.

### Chap. III. The Endemic, or Local Bilious Remittent Fever.

This disease rarely fails to attack the British settler on the Western coast of Africa. It closely resembles the climatorial fever, and is often mistaken for it by the inexperienced practitioner. It requires a different mode of treatment. Mr. Boyle is convinced, from ample experience, that all persons from cold countries "are predisposed or liable, one day or other, to the African local bilious remittent fever," whilst southern subjects, or those of warm climates, are almost entirely exempt from the scourge. The Dutch and Swedes appear to suffer more than those of other European countries as to numbers.

When the rains commence, say at Sierra Leone, the face of Nature quickly changes, and the parched soil teems with vegetation. The inhabitants are soon surrounded by scenery that is alike alluring to the eye and calculated to the rapid diffusion of sickness and death.

"The greater portion, indeed, of the mountainous ridges around Freetown, that is, on the Freetown side, is still covered with bush, and the wreck occasioned by the violence of the tornadoes and the progress of nature, is washed by the deluging showers from the heights down to the bottom, close to the town, where it forms an immense mass of gradually decaying vegetable matter, which continues to disseminate its fatal odours until the setting in of the very heavy rains. When that period arrives, the sun being obscured, the inordinate heat of the surface of the earth is so generally reduced, that evaporation ceases; and the streams resulting from the rains, sweep with so much power that they bear away the vegeto-animal matter, which had previously been collected, and was in a state of decomposition and putridity, or so lessen its quantity and dilute or destroy its pernicious qualities, that its baleful influence sudsides, and no fresh fever cases then appear. This temporary immunity from disease and death is again broken in upon at the approach of the dry season, when, as at the commencement of the rains, the light showers of short duration are disproportionate to the rapidly increasing intensity of the sun; the earth in consequence becomes again heated, and evaporation once more attains its zenith." 126.

The symptoms of the endemic fever are so various and irregular in different cases and under different circumstances, that no concise nosological definition of it can be given; we must therefore make room for Mr. Boyle's more detailed delineation of its symptomatology.

"The first attack of fever, as it is ordinarily observed to occur in the persons of strangers, such generally as are but a short time resident in the colony, although it does not always happen within the first few months, or even the first few years of residence, is characterized by a general decline of natural activity and mental energy; and these symptoms may continue their progress during three, four, or more days, according to the peculiarities of the constitution of the subject attacked, state of the body at the time, and the degree of exposure endured. The disease then begins to unmask itself, either by pains in the limbs, loins, or head, which are not unfrequently accompanied by bilious vomiting; and very soon after, if not at the same time, heat of skin, with a frequent pulse, and a tongue more or less furred, complete the development of the more common symptoms, and remove all doubt as to the existence of the alarming malady. The condition of the bowels forms no criterion by which to judge of the presence or absence of this fever; for they are described by patients, at the onset of the disease, to be in a state that is natural nearly as often as in that of the opposite extremes of costiveness or relaxation. Nor is the skin, except as to its temperature, always to be depended upon as a decisive circumstance on which to determine with respect to the existence of this fever, as the surface of the body is as frequently found to be in a state of copious perspiration as in that of the unpromising converse of dryness and constriction. The pulse, too, though generally more or less above the natural range, is, in other respects, as variable as the winds; it being, in different cases, sometimes hard, yet small, and as it were contracted; at other times, soft, fluttering, or stridulously undulating; and as often regular as irregular, in respect to the intervals between the sensations of its action, as communicated to the fingers and thence to the mind that examines and judges of its state. In some rare cases the pulse is full and bounding. In the latter case a suffused and inflamed appearance of the eyes is generally seen, and the eye-balls, or the lower portion of the forehead, immediately over the orbits, will be complained of as painful; or, if not, minute interrogation will almost always lead to the detection of some fixed or general pain in the head, or to some sensation of weight or confusion in that organ; and, under such circumstances, a strong disposition to sleep will not unfrequently be superadded, attended with sudden starting and an unconquerable degree of restlessness, which effectually prevent indulgence or gratification in the desire. When the pulse, on the contrary, is less developed, the symptoms just described will be either less marked or altogether wanting. In that case pain will be more likely to be referred to the larger joints of the extremities, or to the lumbar portion of the spine, and perhaps to both of these. Occasionally patients are met with, who, though apparently in full possession of all their perceptive faculties, declare they have no pain whatever, expressing themselves as being merely inconvenienced by an indescribable sensation of general *malaise*.

The febrile symptoms are very frequently ushered in by a slight paroxysm, or shivering fit, similar to that of intermittent fever; but which rarely returns during the future progress of the disorder.

Accompanying the premonitory symptoms detailed, yellowness of the skin and eyes, sometimes, also appears ; and this state, when it does appear, is almost always attended with vomiting of green bile, which seldom varies its colour, except as to tint, that being at one time a light, at others, a dark, or bottle green : and in such particular case the early alvine evacuations, at least the evacuations which are procured from the lower bowels, will scarcely ever fail to exhibit a very dark tar-like appearance, and to emit a most offensive odour.

The disease once in march, gradually increases in intensity till it arrives at its critical acmé, which it seldom attains before the eighth, ninth, or tenth day of its duration. In its course many changes are wont to take place ; and two attendant circumstances may be especially noticed as being tolerably uniform in their occurrence, namely, a better and a worse day, alternately ; and a daily exacerbation, which usually commences at about three or four o'clock in the afternoon, and continuing more or less severe all night, ordinarily effects a slow and partial retreat towards the following morning. At this period of the disease the patient will most probably express himself free from pain or suffering, but be greatly fatigued by delirious, or, it may be said, waking dreams, during the previous ten or twelve hours of nervous excitement and mental disturbance. When the patient enjoys this partial respite, he will often recount his fancied adventures, which are never agreeable, but full of apprehension and alarm, and frequently such as, having been attacked by some infuriated animal ; exposure to the fire of a whole army ; tumbling down the most dreadful precipices ; being in the act of drowning ; or about to be tried for his life. In this hallucinated state of the mind, great restlessness is to be observed ; the patient constantly shifting his position in bed, whilst, with his visions still before him, he is complaining, perhaps, of being so firmly bound down that he is unable to move. At times resistance is offered to supposed restraint, and, if not promptly opposed, the patient, maddening under his delusion, will certainly escape from his bed, and, in all probability, dash his way through a window into the streets, or on to a house-top, or into a back-yard, from either of which situations he will frequently extricate himself in a most extraordinary and dexterous manner. This is an extremely dangerous symptom ; it may be said to be almost indicative of certain death. When such an escape has been once effected, or attempted, a repetition of the effort is to be apprehended, and should be well guarded against. The cunning of the patient is remarkable ; for, in the midst of his suffering, his eye is often observed to be fixed on the countenance of the fatigued and dozing nurse, in the hope of detecting her asleep, and of thus finding an opportunity for withdrawing himself from what he considers to be forcible confinement. In such a state of mind no reference will be made by the patient to pain or bodily suffering ; and his answers to questions, on the subject of his feelings, will almost always be, that there is nothing to be complained of but the annoyance arising from cruelty and unkind treatment, which he supposes to be in exercise towards him. Even at this period, within a few hours probably of his dissolution, the patient may be possessed of much physical power, and exert it for a moment, as it were, in a conflict for life. The paroxysm, however, is of brief duration, and is speedily followed by the setting in of symptoms that are unerring in the fatality of their character. The symptoms which immediately precede dissolution, consist of low incoherent muttering, delirium, or perfect calm, scratching at the bed-clothes, or grasping at ideal things, with *subsultus tendinum* ; very frequently, also, involuntary discharges of urine and fæces, attended with lead-cold extremities and hiccup." 131.

Mr. Boyle found out by experience that people labouring under apparently the same symptoms and of similar constitutions, did not bear venesection on the Coast of Africa, by any means so well as in either the East or West Indies. The introduction of a couple of cases, one successful, the other fatal, will sufficiently illustrate the practice pursued by Mr. Boyle in the endemic of Sierra Leone.

" *Case* 1, *from my own Journal, as Colonial Surgeon of Sierra Leone.* William Wilson, a seaman, aged about 25, a well-formed man, of middle stature, was, this morning (17th January, 1828), brought from one of the timber ships up the river: he stated that, the preceding day, he was affected by giddiness and nausea, accompanied by severe pain in the loins, and tottering in the limbs; pulse full and frequent, but not particularly hard; slight redness about the eyes; tongue covered with a yellowish fur; skin in a state of partial perspiration, with great restlessness. The patient's bowels have been moved since his arrival on shore, and he has just vomited a large quantity of yellow bile. He complains now of extremely painful headache; this last supposed to depend upon the vomiting.—A scruple of calomel immediately; and two grains of the same to be continued every two hours after, in the form of pill; the hair to be cut close from the head, which, in addition to the whole surface of the body, when at a temperature above that of the natural standard, and not perspiring, is to be sponged with vinegar and cold water.—At six p.m. eight hours after the first visit, the patient had been greatly purged, but the vomiting and headache were increased; the state of restlessness extremely distressing, with a very hot, dry, and a constricted skin.—Thirty leeches to the forehead and temples, followed by a warm bath, and the application of a blister over the region of the stomach; the two-gr. calomel doses to be each combined with six of calcined magnesia. A little barley-water, gruel, or thin arrow-root, only to be occasionally taken, and that in very small quantities at a time.

18th. Headache gone, but no sleep; vomiting lessened, but not subdued; frequency of pulse and heat of skin greatly reduced.—Saline effervescent draughts occasionally throughout the day; the powders to be continued.

19th. Considerable reduction of the febrile symptoms, but still no sleep; and the draughts and powders are rejected as fast as they are taken.—The powders to be omitted altogether: and, in order to avoid distending the stomach with air, the violence of the effervescence to be permitted to subside previously to swallowing the draughts, which are to be continued. A drachm of mercurial ointment to be rubbed in on the inner side of the thigh every two hours.

20th. Very restless and anxious; had very short slumbers throughout the night, with frightful dreams; skin still less intensely hot; pulse 88, small and not hard; tongue loaded, but moist; no alvine evacuation since yesterday evening; added to which, pressure over the lower bowels produces slight pain.—A seidlitz powder immediately, to be followed by the administration of a mild purgative enema. Mouth not sore.—The mercurial frictions to be continued, and the blister to be dressed with blue ointment.

21st. The patient greatly relieved by the remedies of yesterday; had some sound sleep in the night, but still no appearance of salivation.—Arrow-root, fowl broth, &c., and the other measures to be continued.

22d. Expresses himself perfectly well, but weak; pulse and skin natural; tongue very much loaded; mouth apparently touched, but no saliva: the countenance is demonstrative of much stupor; the whole body, in fact appears torpid; no stool since yesterday morning.—A large blister to be placed over the cervical vertebræ, extending from between the tips of the shoulders to the commencement of the occiput, and a scruple of calomel to be taken immediately; the ointment to be rubbed in every hour.

23rd. The patient decidedly better; complains of pains from his blister, and his mouth, which latter, emits a strong mercurial odour, but saliva does not yet escape from it.—The stomach being again free from irritation, and the system apparently on the very eve of being satisfactorily brought under the mercurial influence, the ointment was left off entirely, and the following medicine directed to be administered every two hours; viz. calomel, one grain; sulphate of quinine, two grains; with six grains of calcined magnesia.

24th. The patient having had a good night, complained only of weakness and irritation, from the state of the mouth and the blistered surface.—To discontinue the powders, and take decoction of bark, with ten grains of sulphate of quinine to each pint of the former. This, together with astringent gargles and occasional mild purgatives, to be continued until recovery of strength.

These means completed the cure, and the patient, perfectly recovered, soon after returned to his vessel.—This, from the first, was a well-marked case of Sierra Leone fever.

*Case 2, from the same Journal.* John Bogue, ætatis 34—admitted on the 19th Jan. 1828. Complains only of weakness and sickness of stomach; had been suffering, during several days on board, from frequent vomiting of green bile. Bowels tolerably free, from the operation of a purgative taken previously to leaving the ship. Pulse now frequent, but weak and tremulous; skin hot, but covered with perspiration; tongue very foul; eyes suffused; no headache; no pain whatever.—Subm. hyd. ℈j. Opii, gr. j., M. divid. in pil. No. iij., statim sumend.

20th. Had been purged slightly in the night; had no sleep; bowels rather irritable; very little other perceptible alteration.—Infus. sennæ, Aq. ammon. acet. āā ℥ss., Sulphas. mag. ʒij., M. statim sumend. postea, Subm. hyd. ℈j., Opii, gr. j., M. ft. pil. No. x. unam quaque secunda hora.

21st. Complains of pain on pressure over the region of the stomach.—A scruple of the submuriate of mercury to be taken immediately, and a large blister to be applied to the affected part.

22nd. Extremely restless and anxious, although the bowels are open and pain is not to be detected, either by examining or questioning the patient; still perfectly rational.—One drachm of mercurial ointment to be rubbed in every two hours; continuing the pills and the other usual auxiliaries in the way of drink, the occasional application of cold affusion, &c.

23d. No improvement of symptoms; on the contrary, the pulse is irregular, and communicates a kind of continuous second stroke; a tremulous thrill hanging, as it were, and returning upon the retiring pulsation: tongue black, dry and contracted, and almost motionless; no stool since yesterday morning; slight nausea, but no vomiting, except immediately after over-indulgence in the beverage directed for his common drink.—A purgative enema to be thrown up immediately, and the medicine to be continued, as directed yesterday.

24th. The patient's bowels open; he had some sleep in the night, but the countenance is greatly altered for the worse: his pulse is very small; he is in a state of stupor, and troubled with hiccough; gums swelled and red, but no saliva flowing.—To omit the mercury altogether; a large blister to the nape of the neck, and to take a third of a wine-glassful of the following mixture every half-hour: Mistura camphoræ, ℔ss., Ammon. carbonat. gr. x., Ether. sulphuric. ʒij. M.

25th. Rallied considerably in the course of yesterday, but is now apparently lost to every thing around him, although the pulse is fuller and softer than it has been for the two or three previous days.—Bottles of hot water to the soles of the feet, which are now somewhat beneath the natural standard, and mustard cataplasms to the calves of the legs; all of no avail: the unfortunate man sunk in the course of the day.

*Dissection.* Cadavre not much emaciated: on laying open the thorax its contents were found to be perfectly healthy, with the exception of old adhesions of the pleura pulmonalis and pleura costalis. On making the usual sections and reflecting back the abdominal parietes in the ordinary manner, the omentum being withdrawn, the whole external aspect of the viscera, *in situ*, had a pale blanched appearance. On laying open the stomach, it was found to be perfectly healthy, containing nothing but a little flocculent mucus, mixed with part of the medicine last prescribed. Liver healthy; gall-bladder distended with dark-green viscid bile ; ducts pervious. Spleen enlarged and easily broken down under the fingers. There was not the slightest appearance of inflammation in any part of the intestines : the duodenum alone contained some of the same kind of mucous matter noticed in the stomach.

It is also to be recollected, in reference to the morbid condition of the parts examined, that the patient was some days ill previous to his having been under medical treatment, thus rendering the appearances after death the more extraordinary." 141.

In addition to cases from his own experience, Mr. Boyle introduces extracts from the journals and reports of various medical officers, who have served on the coast of Africa, and which will furnish valuable documents for those who are doomed to exercise their talents in the same field.

The fourth chapter treats of the "irregular bilious fever" in a very able manner.

The fifth chapter embraces some important subjects—namely, the fatal epidemics which ravaged Sierra Leone in 1823 and 1829. We wish we could afford space for a full analysis of this interesting chapter, as it would afford materials for curious reflection at the present time. The epidemic of 1829 caused discussions similar to those which arose out of the celebrated Bulam fever of Dr. Chisholm, though on a smaller scale. The following short extract will convey some idea of this epidemic.

"The general character and symptoms of the epidemic fever bear so strong a similarity to those of the endemic fever, that it seems to be only necessary to refer to the description of the latter ; and, in addition, to notice separately such points as are peculiar to the former.

The epidemic is more unsparing in its attacks, and more fatal in its consequences, than is the endemic ; and, in fact, no climatorial seasoning gives security against its assaults ; and no practice, hitherto discovered and pursued, has been generally successful in repelling them. It nevertheless, however, so much resembles the endemic in general character, that a broad and vague notion of its qualities may best be formed by supposing those of the endemic greatly aggravated.

With respect to the symptoms, it may be remarked that, as in the endemic fever, there will generally be pyrexia, but rarely so marked or developed, and it will sometimes advance with so insidious a march, as not to attract the particular attention of either the patient or medical attendant. In most cases the action of the pulse will be quickened, and the temperature of the surface elevated ; but it will frequently happen that the patient is first seen in a state of collapse, to which re-action never succeeds. Sometimes pain in the head will be complained of as being very severe ; but more frequently, however, it will be very slight, and not unfrequently altogether absent. Occasionally great giddiness will prevail. There will almost always be pains of the back, loins, or limbs, with pain in the chest, extending along the course of the *œsophagus*, from its commencement even to the stomach ; and this pain will be said to be of a burning description. The state of the tongue varies greatly, it being at one time hard and dry, like a chip, and of a dark brown colour ; the patient being unable to articulate for want of saliva. At other times the tongue will have a white centre, with edges of a bright red ; and, still more commonly, in the worst cases, the tongue will be altogether without fur, and of a deep blood-red colour, and either very much enlarged and sponge-like, or elongated and contracted at the tip. There is generally thirst and a desire for cold liquids. The bowels are, for the most part, deranged ; sometimes constipated, and sometimes, on the other hand, there is slight purging, attended with griping or tenesmus. The red appearance of the tongue, and the pains of chest, back, loins, or lower extremities, may be considered as the truest characteristics of the existence of the disorder." 203.

It is not a little remarkable that a grand jury of tradesmen was set up in June, 1829, to investigate the remote cause of the epidemic, and its mode of propagation!! This jury, after mature deliberation, no doubt, came to the following conclusions—1st, that the then unhealthy state of Freetown was to be attributed to the landing of slaves from prize-ships in the centre of the town—secondly, to dead horses and cattle thrown into the water—and, thirdly, to the existence of certain slaughter-houses too near high water mark. All these allegations are denied by Mr. Boyle; but his refutation of them need not detain us here. The opinions of the grand jury died quickly away in consequence of a new idea being started, which occupied the attention of all classes. This was the importation of the epidemic through the medium of His Majesty's ship Eden. It was ascertained that previously to the Eden's arrival in Sierra Leone, the only complaints which prevailed in that vessel, were diarrhœa. But she was found to be rather dirty, and the medical examination considered that she brought the seeds of the fever which broke out in her at Freetown, from Fernando Po. Other sources of the epidemic were now searched for and found in certain schooners or slavers that had arrived in the colony about the time the epidemic broke out. A full investigation proved that no other complaint prevailed in these slavers besides *ulcers!* But we cannot follow our author through all the absurd creeds of importation which prevailed in Sierra Leone at the above period. One would suppose that the colony in question ought to be the last in the world to accuse other countries of exporting diseases into that quarter! We cannot resist, however, the introduction of a document drawn up by a mixed commission, partly civil and partly medical, sent to the Bullom shore, to ascertain whether the epidemic of Sierra Leone was confined to that port alone.

"*Narrative of a Visit made to the Bullom Country for the purpose of acquiring Information respecting the Epidemic Fever of* 1829.

Apprehensive that political motives, in regard to the character of the country, and with respect to the effect which might be produced on their trade, might induce the natives to hesitate to make us acquainted with all they knew, we took with us a few trifling presents for the purpose of rewarding truth. Thus furnished, we proceeded, at an early hour, on the morning of the 26th of July, to Dalla Moodie's residence, which is situated in a native town, named Medina, and is distant from Freetown about nine miles. On explaining the object of our visit, that chief expressed his readiness to give us all the intelligence he possessed, and we soon proceeded to business. Several messengers from the interior were with Dalla Moodie at the time, and we had speedily the satisfaction of finding that there was no lack of the information sought for. In order to facilitate our inquiries, we took with us a chart. Dalla Moodie soon shewed that he understood the relative situations of the four quarters of the globe; and, with the assistance of that knowledge, he rendered clear and comprehensive his explanations of places and local situations, and all such matters. When his recollection failed, he referred to manuscript writings in Arabic, and to persons around him from the interior. In describing his communications, no attempt will be made to adhere to his particular language, but the facts detailed will be faithfully conveyed. The information with which he furnished us was to the following effect:—

'In cutting the last crop of rice in November and December, 1828, the usual heavy tornadoes, which generally blow with great force at that season in the north-easterly direction, did not prevail. This circumstance was noticed by the natives generally; who, on the absence of those winds, at the periods above stated, always apprehend that the ensuing season will be particularly unhealthy. During the prevalence of the harmattan winds the air is impregnated, in a considerable degree, with an inpalpable sand; and, after their cessation, the leaves of trees, &c. are found covered with it. These leaves are ordinarily cleared of the sand by the succeeding tornadoes, which set in about the month of April; but, if the latter are not sufficiently strong, the sand, previously so deposited, remains; and this circumstance, whenever it occurs, is universally acknowledged, amongst the natives, as a certain sign that the following season will be unhealthy. This particular omen was observed by the surrounding people this year, who, in each town, consistently with their accustomed habits on such threatening occasions, contributed, according to the means of the respective individuals, a sum, or rather goods equal to a certain amount, for alleviating the anticipated necessities of their poorer brethren.

After cutting the rice, the stubble is always left on the ground, and, of course, becomes gradually decomposed, and emits more or less extensive exhalations of vegetable *malaria;* for the grain (the rice) is generally grown in low swampy situations. The ordinary, and, indeed, necessary consequence of this occurrence is sufficiently injurious, but it was greatly aggravated this year by premature setting in of the tornadoes, and the unusually early fall of frequent showers of rain. This irregularity in the seasons, in addition to increasing the usual malaria, did not allow sufficient time for burning the bush which had been cut down on large tracks of land, for the purpose of their being sown with rice; and the bush, being left exposed to the atmosphere and the weather, the natural consequence was, that it soon became so many masses of putrid vegetable matter, constantly emitting the most noxious and fatal exhalations, which were generated by the sun and partial rains, and dispersed according to the frequency and violence of the tornadoes. The natives thus accounted for the extent and fatality of the disease; and their reasoning appears to be convincing.'

'The sickness, with which the colony of Sierra Leone and the surrounding country have been so fatally afflicted, first made its appearance in Sangarrah, a country in the interior, about thirty days journey, in a north-east direction from Medina, the residence of Dalla Moodie. The fever broke out at Sangarrah about Christmas, 1828, and more chiefs have suffered from it there within a few months than were ever before recollected to have suffered, from a similar cause, in as many years. Sangarrah is a low marshy country, covered with jungle. Bantoe, lying to the south-west of Sangarrah, is also said to have suffered exceedingly. Out of twelve men sent out in March to hunt elephants, only one returned alive, the others having died in the forests from the effects of the unhealthy condition of the atmosphere. Between Sangarrah and Foutah Jallon, whole villages have been nearly depopulated.

From Laheer, and down through the Mandingo country, travelling in a south-west course to Fouricaria and Melicorree, the number of chiefs that have died is unprecedented; and, amongst the people generally, the loss has been immense. Much sickness prevailed in the Foulah country; but, owing to its being better cultivated, and composed more of hill and valley, it has not suffered to so great an extent as Sangarrah, where the fever broke out.

The symptoms at the commencement of the attack were, a sensation of cold, followed by pains in the chest and region of the stomach, and sickness, with occasional vomiting; pains in the head and back, attended with loss of appetite. Matter ejected by vomiting was sometimes yellow and sometimes black. Hot skin and great thirst; state of tongue not known; no sleep; delirium and sudden starting. Fires were much used in the houses, and, as the natives believe, with great advantage. To promote perspiration appears to have been a great object. Cathartics freely administered. No other treatment for the purpose of cure was very clearly expressed, except with reference to the restoration of strength.

The number of inhabitants at the village of Yongroo, in the neighbourhood of Medina, was not accurately known ; but Dalla Moodie was himself aware of four deaths having occurred there. His own town, Medina, was, and has been healthy.'

Such was the result of the visit to the Bullom Shore ; and, from further and rather extensive inquiries of the natives from various parts of the main land, it was fully ascertained, that a fatal epidemic fever had prevailed to great extent in the following countries amongst their respective tribes—namely, Bullom, Porto Logo, Rokelle, Scarcies, Mellacoree and Foolah. Those from whom these statements were obtained, were unanimous in the opinion, that the disease proceeded from the eastward, or, agreeably with their own account, from where the sun rises.

'From all the descriptions given, and the facts obtained, relative to the comparative mortality in the vicinity of the colony, it appears that it was greatest in the neighbourhood of Porto Logo, and down to the swampy tongue of land which separates Porto Logo from the small Scarcies ; thence along the bank of the large and small Scarcies,* and across the country to Mellacoree and Foreecarreah. The banks of the Large Scarcies and of the Mellacoree, in particular, are much intersected with creeks lined with mangrove, and generally ending in swamps, which are always overflowed in the rains ; and, after their departure, emit the most offensive and most unhealthy odour.

If the people from any of the countries named were asked what the sick persons complained of, they replied, of fever—'his skin hurt him, his head hurt him,' and 'his middle (his back and loins) hurt him.' Some supposed the disease was originated by witchcraft ; the witch causing a poisonous atmosphere, the atmosphere still the immediate promoter, to rise out of the ground and to act on those whom she disliked.

From messengers from Timbo, in the beginning of August, it was ascertained that an epidemic prevailed there twelve months previously to that date, and that it had then ceased only four months. The complaint was described by these people as being attended with pains in the head, back, and loins, with a hot skin and black tongue. In the advanced stage of the complaint the gums and tongue exhibited the colour of blood ; and, it was added, that some threw up a black fluid like a mixture of powder.

The Timbo people believed the sickness took its origin in the attempt at clearing a swamp in the neighbourhood of Timbuctoo, and that it thence spread to Jenni, from Jenni to Foota Tauro, and thence to Footah Jallon. It was also understood to have spread in the neighbourhood of Tamassoo, Tambacca, and Kissy Kissy.'

The foregoing information appears to be so conclusive, as to the origin and original nature of the disease, that further discussion upon those points must be unnecessary. That there are differences in the accounts given by the natives is true, but they do not amount to discrepancies of such a magnitude, as at all to invalidate or shake the one leading supposition and conviction—that the epidemic fever, which raged so fatally in Freetown in 1829, was immediately caused by peculiarities in the seasons—originated in the interior—was borne to Free-

---

* "It is important here to remind the reader it was at the Scarcies that the second and third cases of epidemic were contracted, which occurred on the 4th and 9th of May in Freetown."

town by the north-east winds—and, in its primary and true character, was not contagious." 258.

The good folks of Freetown, however, from the Governor (Major Ricketts) downwards, became panic-stricken, and "sauve qui peut," was the order of the day, notwithstanding the official opinions of all the medical officers of the colony that the epidemic was non-contagious!

"That great apprehensions of contagion prevailed, and that personal safety was much consulted, is beyond all question; and the attempt made to stay the fear was unfortunately ineffectual. The facts are, that, soon after the publication of the above medical opinions, the two army medical officers were reported sick; fever cases became more general; a few individuals retired from the colony; the governor went to the military barracks; and many of the inhabitants laboured under the dangerous influence of a prevailing panic. Misstatements went abroad respecting the state of the gaol; in consequence of which, the then Chief Justice declined to hold the sittings of Quarter Sessions in the court-house situate over that establishment, and exhorted the Grand Jury, in a public speech, not to enter the gaol without each juryman being supplied with a bottle of chlorate of lime, which, in procession, they were to sprinkle in all directions.

In this state of circumstances it became no easy matter to brave the besetting difficulties amongst a deserted and consequently disheartened community; for, as it is with soldiers in the field of battle, so is it with the subjects of a community when surrounded by sickness; they will quail, fly, or sink in despair, if the chief absent himself from his post." 268.

With this curious extract we must close our imperfect notice of this valuable volume. To all army and navy surgeons, who may be destined to visit tropical climates generally, and the African Coast in particular, Mr. Boyle's volume is absolutely indispensable. And, indeed, we strongly recommend it to the perusal of our brethren in all countries.

